# Survey Dataset Supporting Blue Circular Economy Strategies for Bivalve Shell Waste Valorisation

**DOI:** 10.1016/j.dib.2026.112929

**Published:** 2026-06-03

**Authors:** Poliana Bellei, Fernanda Caroline Magalhães, Maria Paula Mendes, Rui Vasco Silva, Manuel Francisco Costa Pereira, Ana Paula Soares Dias, Marte Jenssen, Runar Gjerp Solstad, Mahdi Kioumarsi, Inês Flores-Colen

**Affiliations:** a CERIS, DECivil, Instituto Superior Técnico, University of Lisbon, 1049-001 Lisbon, Portugal; b CiTUA, DECivil, Instituto Superior Técnico, University of Lisbon, 1049-001 Lisbon, Portugal; c CERENA, Instituto Superior Técnico, University of Lisbon, 1049-001 Lisbon, Portugal; d Nofima, The Norwegian Institute of Food, Fisheries and Aquaculture Research, 9019 Tromsø, Norway; e Department of Built Environment, Oslo Metropolitan University, 0166 Oslo, Norway

**Keywords:** Waste reuse, Aquaculture, Invasive, Oyster shell, Dataset

## Abstract

The circular economy advocates for a fundamental shift, focusing on the reuse, recycling, and valorization of waste to keep materials in use for as long as possible, thereby reducing the need for new resources and minimizing waste. In this context, the concept of the Blue Circular Economy emerges, emphasizing the preservation and sustainable use of marine and coastal resources.

This paper presents and explains a dataset collected through a survey administered in two regions: Algarve-Portugal, and Oslo-Norway. The aim was to address the existing data gap on the production, consumption, and disposal of bivalve shells from aquaculture, as well as to assess stakeholders' perceptions of the reuse and circularity of products. In Portugal, data collection focused on analyzing the mollusk supply chain in the Algarve region and assessing the potential for oyster shell reuse within the country. In Norway, data collection targeted the construction industry and academia to understand their perspectives on the reuse of shells in building applications.

The dataset comprises responses from 112 voluntary participants from different countries, categorized into three main groups. The questions addressed the quantity of bivalves produced and consumed, shell disposal practices, perceptions regarding the potential reuse of this waste, and the level of awareness of public policies promoting circular economy strategies. Upon completion of the survey, respondents were given the opportunity to receive a summary of the study findings by voluntarily providing their e-mail address in a designated field of the survey.

The dataset integrates quantitative data from online and local face-to-face surveys across different groups. This dataset provides a valuable resource for stakeholders in the aquaculture and construction sectors seeking to explore the potential of this biomaterial to valorise waste into innovative products, as well as for public authorities aiming to support the transition towards a circular economy*.*

Specifications Table

**Table:** **General information about the dataset**.

Subject	Social Sciences
Specific subject area	*Production, consumption and disposal of bivalve shells and their social, environmental and economic impacts*
Type of data	Excel spreadsheet.
Data collection	These survey items were taken from previous research. The survey was developed as part of the first activity of the Shellter project and of the master's thesis titled "Blue Circular Economy in the Algarve: Reuse and Valorization of Shellfish Shells." The first data collection and face-to-face interviews took place from 15 to 21 April 2023 in the southern region of Portugal, in the cities of Olhão, Faro, and Culatra Island. Participants included producers, intermediaries, and construction companies. At a later stage, in Oslo, Norway, data collection took place from June 19 to 21, 2024, during an international conference and the first author’s internship. In Norway, the target groups were civil engineering researchers and industry professionals.
Data source location	Portugal (including Portuguese participants) and Norway (including participants from Europe, Asia, Africa, and North America).
Data accessibility	Repository name: Mendeley dataset website Data identification number: doi:10.17632/cyyy6mwktp.1 Direct URL to data: https://data.mendeley.com/datasets/cyyy6mwktp/1
Related research article	Magalhães, F. C., Bellei, P., Flores-Colen, I., & da Costa, E. M. (2024). Blue Circular Economy - Reuse and Valorization of Bivalve Shells: The Case of Algarve, Portugal. Recycling, 9(2), 27. DOI: https://doi.org/10.3390/recycling9020027

## 1 Value of the Data

• **Comprehensive Coverage:** This dataset provides a thorough overview of the bivalve shells production, namely oyster shells, by quantifying shell-waste streams, including consumption indicators such as mortality rate, unsold volumes, and current disposal routes. This detailed coverage is valuable to researchers studying waste management and resource reuse across diverse geographical and cultural contexts.
• **Engagement with Stakeholders:** The dataset offers a platform for engaging stakeholders involved in the bivalve shell circuit. By analyzing the data, researchers and professionals from the aquaculture and construction industry, and other industries can better understand the needs and perspectives of these stakeholders, facilitating more informed and collaborative approaches to waste management and product development.
• **Addressing Environmental Challenges:** The data offers valuable insights into the waste management challenges associated with bivalve aquaculture, particularly the disposal of shells. By analyzing these components, professionals from the aquaculture industry and other industries can better understand the environmental impact and explore sustainable waste management practices, aiming to reduce reliance on landfills and incineration.
• **Identifying Opportunities for Circular Solutions:** By highlighting the potential for repurposing bivalve shells, this dataset supports the identification of blue circular economy strategies within the aquaculture sector. It enables researchers and stakeholders to explore innovative uses for shell waste, paving the way for technologies and processes that maximize resource value and reduce environmental impact.

## 2 Background

This article supports an original study that explores how the principles of the Blue Circular Economy can mitigate waste and pollution by reducing improper disposal of aquaculture waste in the Algarve, Portugal [1,2].

The southern region of Portugal, the Algarve, is one of the country's largest producers of bivalves, thanks to favorable production conditions and tourism [3,4]. In Norway, the Pacific oyster is an invasive species that threatens the local ecosystem by spreading diseases among native bivalves [5]. Both countries are seeking strategies to manage the high production of shell waste.

**Fig. 1:**
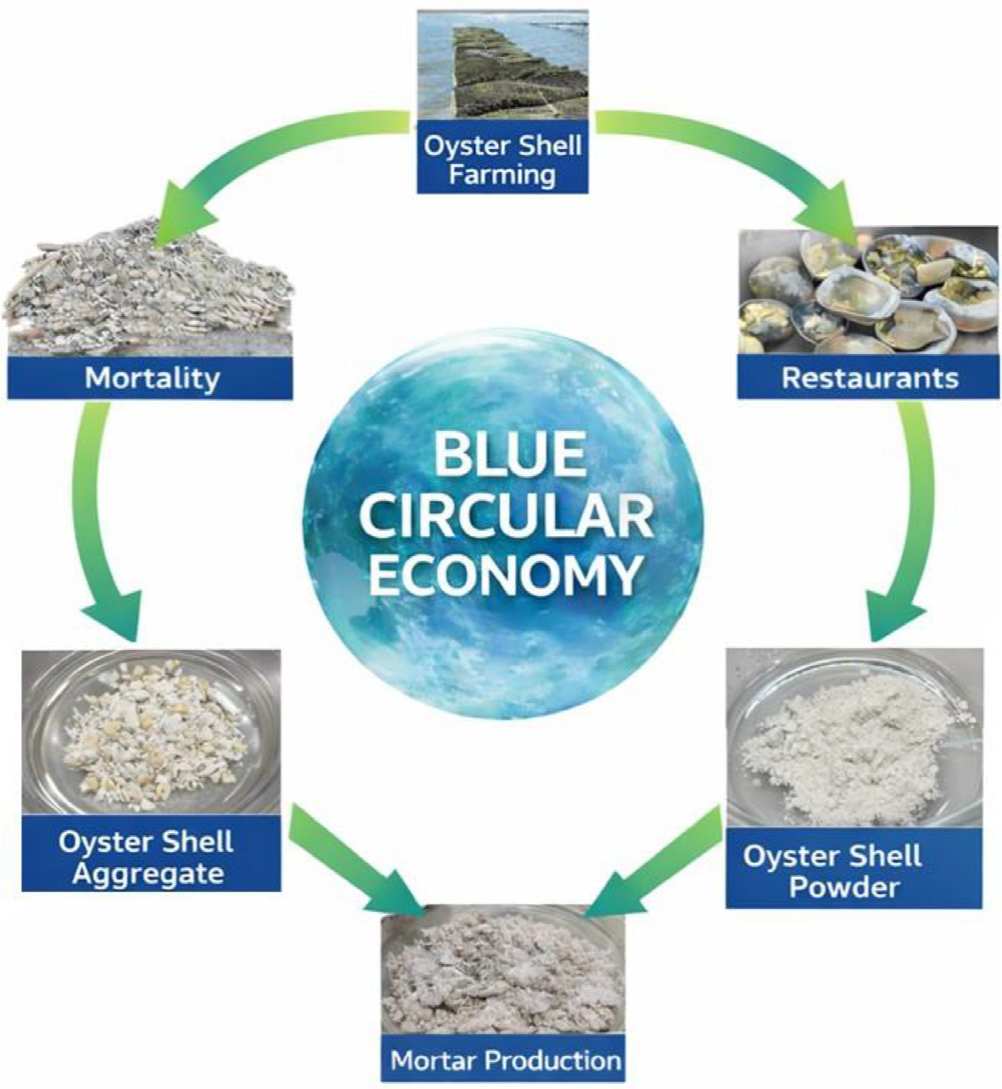
Representation of the Blue Circular Economy model for oyster shell waste valorisation.

The rapid growth in global marine bivalve production (global aquaculture production grew by over 600% between 1990 and 2020 [6]) underscores the need to examine the components of aquaculture and their impacts, particularly waste generation. Bivalve shells are often perceived as troublesome waste rather than valuable commodities, leading to their disposal in landfills or incineration. Therefore, promoting the circularity of these waste products (e.g., oyster shells as construction materials, Fig. 1) is crucial for achieving environmental, economic, and socio-cultural benefits [7,8], as advocated by the FAO's Blue Transformation initiative [9].

### 2.1 Data Description

Data processing was conducted through two approaches: (i) quantitative response, which involved handling the results and creating charts and tables using Excel; and (ii) descriptive response, where stakeholders were encouraged to indicate whether they were aware of any oyster shell reuse practices.

A total of 112 participants responded, of whom 75% were involved in the first phase of data collection (15 to 21 April 2023) in the southern region of Portugal, specifically in the cities of Olhão and Faro, and on Culatra Island. The remaining 25% of responses were collected in Oslo, Norway, from June 19 to 21, 2024.

The dataset is in an Excel file, with seven sections. The survey was structured into three distinct target groups:

• Group 1 – Producers: Aquaculturists, Scrubbing plants or Distributors, Associations or Cooperatives;
• Group 2 – Intermediaries: Restaurants, Hotels, and End Consumers;
• Group 3 – Stakeholders: Researchers and Industry (Construction).

An overview of the database content presented in the Excel file is given in Table 1.

**Table 1:** Database content presented in the Excel file.

Sections	Title	Parameter Content
1	Summary	This tab outlines the purpose of the Shellter project and the survey, and briefly describes the content and organization of the file
2	1. Survey	This tab contains a comprehensive list of survey items for all three groups of participants
3	2. Target Groups	This tab presents the breakdown of survey groups by category and includes the circuit of bivalves sourced from the database
4	3. Group 1	This tab contains the questions and corresponding responses from Group 1
5	4. Group 2	This tab contains the questions and corresponding responses from Group 2
6	5. Group 3	This tab contains the questions and corresponding responses from Group 3
7	6. Further Information	This tab lists the main references and web pages related to the project participants, including links to publications and the dissemination of results

**Fig. 2:**
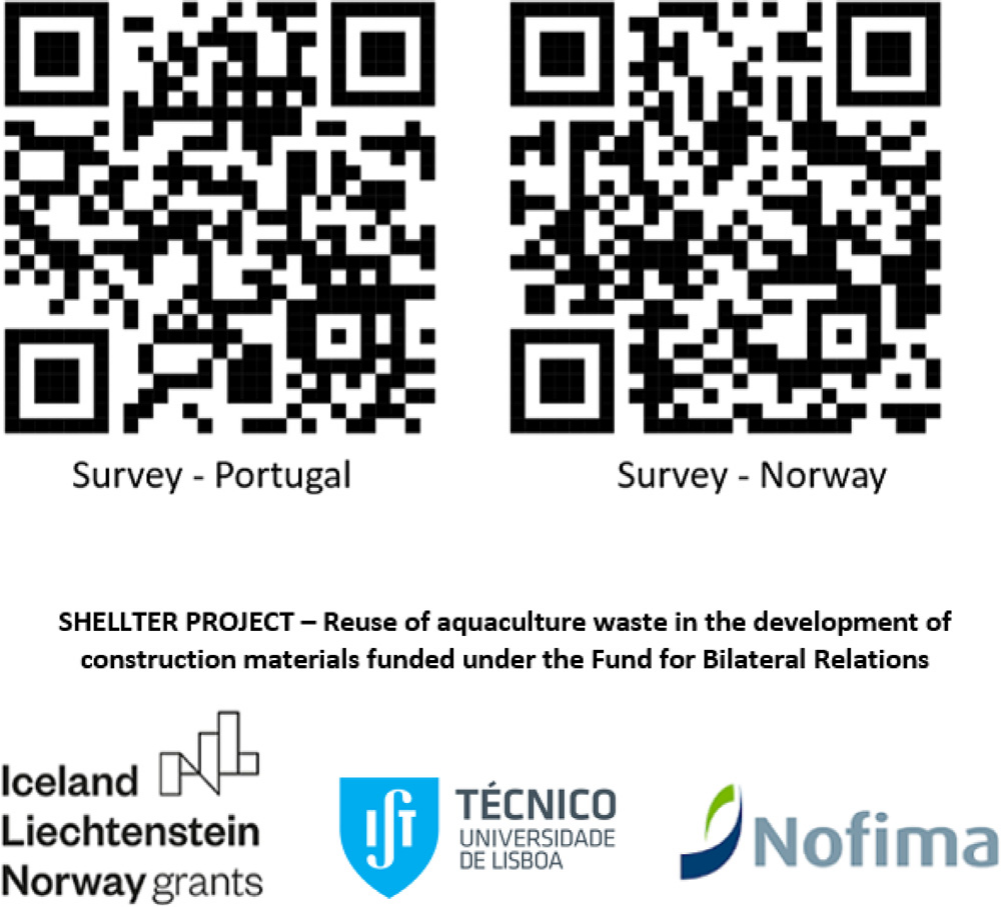
Poster displayed to survey participants.

Among the participants, there was a distribution of responses as follows: 42 were collected via digital surveys (Google Forms), 11 were acquired via telephone application, and 59 were conducted by face-to-face survey. All obtained responses were standardized and harmonized within the database. The digital survey was distributed both in person and by email, using a QR code that provided access to the online form (Google Forms). The survey was developed in both English and Portuguese for distribution in Portugal and exclusively in English for distribution in Norway (Fig. 2). Of the total participants, 76% are from Portugal, with the remaining 24% distributed across the continents of Europe, North America, Asia, and Africa.

Participants in Groups 1 and 2 are all from Portugal. However, in Group 3, only 10% of the participants reside in Portugal, while the remaining 90% are distributed across the countries (Norway, China, Germany, Italy, the United Kingdom, Morocco, Colombia, France, the Netherlands, South Africa, and the United States) covered by the survey.

## 3 Experimental Design, Materials and Methods

The dataset was compiled as part of a master's dissertation with the aim of exploring how the principles of the Blue Circular Economy can mitigate waste and pollution by reducing the inappropriate disposal of aquaculture waste. The work was aligned with the objective of the Shellter project, a collaboration between the Instituto Superior Técnico, University of Lisbon and the Norwegian company Nofima, with funding from the EEA Grants Bilateral Relations Fund, which seeks to evaluate the potential for integrating oyster shells into the production of building materials.

**Fig. 3:**
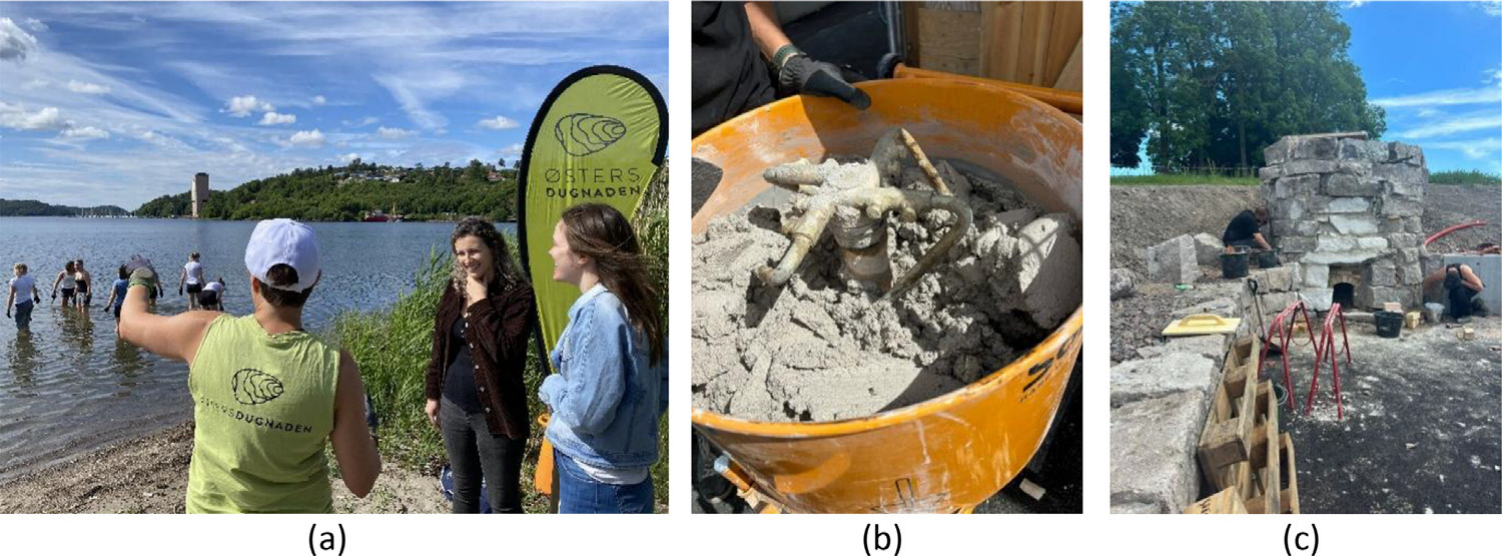
The collection of invasive oysters on the sea (a); preparation of shell-based mortar (b); and the rendering of the oven used for calcining the collected shells (c).

The survey was conducted in two stages: the first in Portugal from April 15 to 21, 2023, and the second in Norway from June 19 to 21, 2024, during an international conference and an internship under Shellter project. The data collection in the first stage (Portugal) focused on the development of the Bivalve Circuit and a SWOT analysis of the potential of the residues (both are available online [1,2]). In the second stage (Norway), the focus was entirely on the industrial sector and researchers to understand their perception of the potential for reusing shells in the construction industry. A local technical visit to the Stokke community was also conducted to observe the use of invasive oyster shells in construction (Fig. 3).

Through local face-to-face survey crucial gaps regarding the cultivation, consumption, and disposal of bivalves were fulfilled.

In the survey, the **first group** identified was the producers, classified into three main categories: a) Independent Bivalve Producers: With relatively low production, these producers cultivate smaller quantities of bivalves independently, supplying them to the local or regional market. b) Producers Associated with Cooperatives or Purification Facilities: These producers have access to shared resources, such as infrastructure, storage, and marketing, through partnerships with cooperatives or purification facilities. They serve both local and regional as well as international markets, with average bivalve production. c) Export-Oriented Producers: With medium to high production, these producers focus on export, dedicating a significant portion or all of their production to markets outside Portugal. They are typically involved in processing activities and have a broader commercial focus.

The **second group** was the intermediaries, including end consumers, restaurants and hotels. Finally, the **third group**, stakeholders, was targeted at researchers in civil engineering and industry.

The questions were designed to investigate how the bivalve circuit impacts social, environmental, and sociocultural dimensions, with questions tailored for each specific group. For the first group, questions addressed aspects related to production quantity, marketing, bivalve disposal, and perceptions of the value of the generated waste. The second group was questioned about bivalve consumption and disposal, perceptions of waste value, and awareness of regulations promoting proper disposal. The third group focused on the characteristics of the shells, including reuse practices, waste repurposing types, and the integration of these materials into construction.

The Shellter project aimed to repurpose aquaculture products to develop construction materials. At the Instituto Superior Técnico (IST), as part of this project, a master's dissertation on the valorization of bivalve shells and a doctoral thesis focused on testing the use of oyster shells in rendering mortars were completed [[10], [11], [12], [13], [14]]. Furthermore, another master’s dissertation is underway as part of another ongoing project the completed BlocOyster project, which aimed to explore the oyster shell residue and other bivalve residues from aquaculture as an industrial product for companies committed to sustainability, in this case through lightweight blocks*.*

## Limitations

The uneven distribution of respondents across the survey categories affected the data's representativeness. The sample size was also restricted, which limited the overall perspective on the topic. The survey interviewed only one stakeholder group, focusing on the construction sector.

## Ethics Statement

The authors of this article are aware of and agree with the ethical statements of this journal*.*

## CRediT Author Statement

**Poliana Bellei, Fernanda Caroline Magalhães:** Conceptualization, Methodology, Validation, Formal analysis, Data curation, Writing – original draft, Visualization; **Maria Paula Mendes, Rui Vasco Silva, Manuel Francisco Pereira, Ana Paula Soares Dias, Marte Jenssen Mahdi Kioumarsi:** Conceptualization, Methodology, Validation, Data curation, Writing – review & editing; **Runar Gjerp Solstad:** Conceptualization, Methodology, Validation, Writing – review & editing, Funding acquisition; **Inês Flores-Colen:** Conceptualization, Methodology, Validation, Writing –review & editing, Funding acquisition, Project administration.

## Data Availability

Mendeley DataShellter project database - The Potential Use of Waste from Shellfish Aquaculture in the Production of New Construction Materials (Original data).
